# Hypertension in older patients, a retrospective cohort study

**DOI:** 10.1186/s12877-016-0316-0

**Published:** 2016-07-19

**Authors:** C. G. H. Blok, M. A. J. de Ridder, K. M. C. Verhamme, P. W. Moorman

**Affiliations:** Department of Medical Informatics, Erasmus Medical Centre, P.O. Box 2040, 3015 CA Rotterdam, The Netherlands

**Keywords:** Hypertension, Aged 80 and over, Age factors, Electronic health records, General practice

## Abstract

**Background:**

It is unknown to what extent General Practitioners (GPs) manage hypertension (HT) differently in older patients, as compared to younger age groups. The purpose of our study was to compare HT management in older patients to younger age groups.

**Methods:**

We performed a retrospective cohort study of patients of 159 GP's practices in the Integrated Primary Care Information (IPCI) database. The study period lasted from September 2010 through December 2012.

The study population consisted of all patients aged 60 years or older with at least one blood pressure (BP) measurement during the inclusion period, without pre-existent HT, diabetes mellitus (DM) or atherosclerotic cardiovascular disease at time of study start.

Study outcomes were a diagnosis of HT within one month after cohort entry and the use of antihypertensive medication within 4 months after cohort entry in HT diagnosed patients.

We compared the incidence of outcomes between the age groups, stratified by systolic blood pressure (SBP). Logistic regression analysis was used to assess the influence of age-adjusted SBP Z-scores, age and gender on the outcomes.

**Results:**

We included 19,500 patients from 159 GP’s practices of whom 1,181 (6.1 %) were newly diagnosed with HT. Corrected for age-adjusted SBP, older patients were less likely to be diagnosed with HT (odds ratio per year age increase 0.98, *p* < 0.001). Corrected for age-adjusted SBP, no significant effect of age on the probability of treatment in newly diagnosed HT patients was observed (*p* = 0.82).

**Conclusions:**

This study showed that GPs are less inclined to diagnose HT with increasing patient age, but do not withhold treatment when they diagnose HT in older patients.

## Background

Evidence on the effectiveness of treatment of hypertension (HT) in patients of 80 years and older is conflicting [1–4]. Traditionally older patients were ignored in Dutch guidelines as patients up to 65 years old were treated according to their risk estimate of atherosclerotic cardiovascular disease, for which blood pressure (BP) was one of the criteria [5]. For older patients however, the general practitioner (GP) had to rely on his/her clinical judgment, which varied widely as to when it was appropriate to start treatment [6].

In 2008 the Hypertension in the Very Elderly Trial (HYVET) [1] indicated that treatment of HT in patients of 80 years and older was beneficial on mortality from stroke and overall mortality. There is evidence that high BP is not associated with a higher risk of death in the frail [3], but further analysis of the HYVET study showed that both frail and fitter patients appeared to gain from antihypertensive treatment [5].

In 2012 the Dutch cardiovascular risk management guidelines [7] set systolic blood pressure (SBP) target values below 150–160 mmHg in older patients. Similarly, guidelines of the British National Institute for Health and Care Excellence (NICE), the European Society of Hypertension (ESH) and the European Society of Cardiology (ESC) recommended SBP target values below 150 mmHg in patients over 80 years old [8, 9]. However, there is evidence that compliance to these revised guidelines is incomplete [10], particularly in older patients [11, 12] and a recent review suggested that indeed, each patient’s individual clinical condition may need to be taken into account for optimal HT treatment [13]. In practice, other than patient-related barriers, e.g. doctor or system related barriers, whether they are appropriate or inappropriate, may play a role in incomplete guideline compliance.

In light of the conflicting evidence and lack of information whether GPs manage HT differently in older patients, we wanted to investigate how HT is diagnosed and treated in this age group, as compared to younger patients.

## Methods

### Setting

We conducted a retrospective cohort study within the Integrated Primary Care Information database (IPCI); a longitudinal observational dynamic database which contains the complete electronic medical records of the patients of 159 GP’s surgeries in the Netherlands during the full study period. In the Dutch health care system, patients are registered with a single GP who acts as a gatekeeper for and receiver of information from secondary care. Details of the database have been published elsewhere. [14, 15]. In brief, the database contains anonymous longitudinal data on demographics, symptoms and diagnoses (in coded and free text format), referrals, laboratory findings, discharge letters, and drug prescriptions. To maximize completeness of the data, GPs participating in the IPCI project are not allowed to maintain a system of paper-based records besides the electronic medical records. To enhance data quality, GPs are encouraged to encode their assessments, which is also facilitated by their record systems. The IPCI database system complies with European Union guidelines on the use of data for medical research and has been proven valid for pharmaco-epidemiological studies [15].

### Study population and study cohort

Within the IPCI database we defined the study population as all patients who were present in the database during the entire period of the 1st of September 2009 to the 31st of December 2012.

Within this study population, we defined a cohort in which we included all patients aged 60 years or older, with at least one BP measurement between1^st^ of September 2010 and August 31th 2012 (the inclusion period). The date of inclusion was defined as the date of the first BP measurement within the inclusion period. Follow-up for this cohort started upon first BP measurement until maximum four months after inclusion. We excluded patients who, prior to their first BP measurement, had already been diagnosed with HT, diabetes mellitus or atherosclerotic cardiovascular disease, including atrial fibrillation. We furthermore excluded patients who had received antihypertensive medication in the 12 months preceding the first BP measurement. In Appendix 1, we provide a more detailed overview of these criteria.

### Covariates

Age at date of inclusion was considered and stratified in 3 categories namely 60–69, 70–79 and 80+ years old. SBP was retrieved from the patient’s measurement file and categorised into 5 strata namely lower than 140 mmHg, 140–159 mmHg, 160–179 mmHg, 180–199 mmHg and higher than 200 mmHg.

### Outcome parameters

The two outcomes of interest were: diagnosis of HT and start of anti-hypertensive treatment. Diagnosis of HT was based on disease codes and the complete medical record was searched for International Classification of Primary Care (ICPC) codes of HT: K86 (essential HT without organic damage), within one month after the first BP measurement (ie inclusion). Treatment for HT was based on prescriptions of antihypertensive drugs (thiazide diuretics, calcium channel blockers, renin-angiotensin-aldosterone system (RAAS) inhibitors and beta blockers) within 4 months after the first BP measurement that preceded the HT diagnosis. Details about the relevant Anatomical Therapeutic Chemical (ATC) codes are described in Appendix 2.

### Analysis

Proportions (with Wilson 95%CI) of patients with a new diagnosis and treatment of HT were calculated stratified by age group and SBP category. Within each SBP category the age-outcome relation was tested using linear-by-linear association.

SBP Z-scores were calculated using sex and age specific reference values from the World Health Organization (WHO) [16]. For example: for a 65-year-old woman the reference mean SBP is 151 mmHg, with a standard deviation of 23. If her SBP is 151 mmHg the SBP Z-score would be 0 and if her SBP is 174 mmHg, her SBP Z-score would be (174–151)/23 = 1.

The possible influence of these SBP Z-scores, age and gender on the probability of HT diagnosis was tested using logistic regression. If SBP Z-score and age or gender had a significant contribution to the model, the significance of their interaction was tested.

In patients with an HT diagnosis, influences on the probability to get treatment were tested similarly.

## Results

### Patient characteristics

Our cohort consisted of 19,500 patients aged 60+ with at least one BP measurement and without a diagnosis of HT, DM or arterial cardiovascular disease or antihypertensive medication prior to the first BP measurement. The source population comprised of 131,545 patients aged 60 years or older.

Sixty one percent were 60–69 years old, 28.2 % were 70–79 years old and 10.8 % were 80 years or older. The percentages of women in these age groups were 55.7, 57.9 and 64.0 % respectively. Table 1 shows SBP values, by age group and gender.

**Table 1:** Systolic Blood Pressure by gender and age; mean SBP with standard deviation; percentages above 140 mmHg and 160 mmHg

Age	N	Gender	SBP (mmHg)		SBP > 140 mmHg	SBP > 160 mmHg
			Mean	SD		
60–69	5270	male	142	19.9	55.8 %	20.7 %
	6619	female	141	20.7	53.8 %	20.8 %
70–79	2316	male	145	20.8	63.1 %	26.4 %
	3182	female	146	21.2	65.1 %	28.3 %
80 +	761	male	146	21.6	64.5 %	27.9 %
	1352	female	149	22.2	71.1 %	32.0 %

## Outcomes

### Percentages of patients with diagnosis of hypertension, by age and systolic blood pressure

**Fig. 1:**
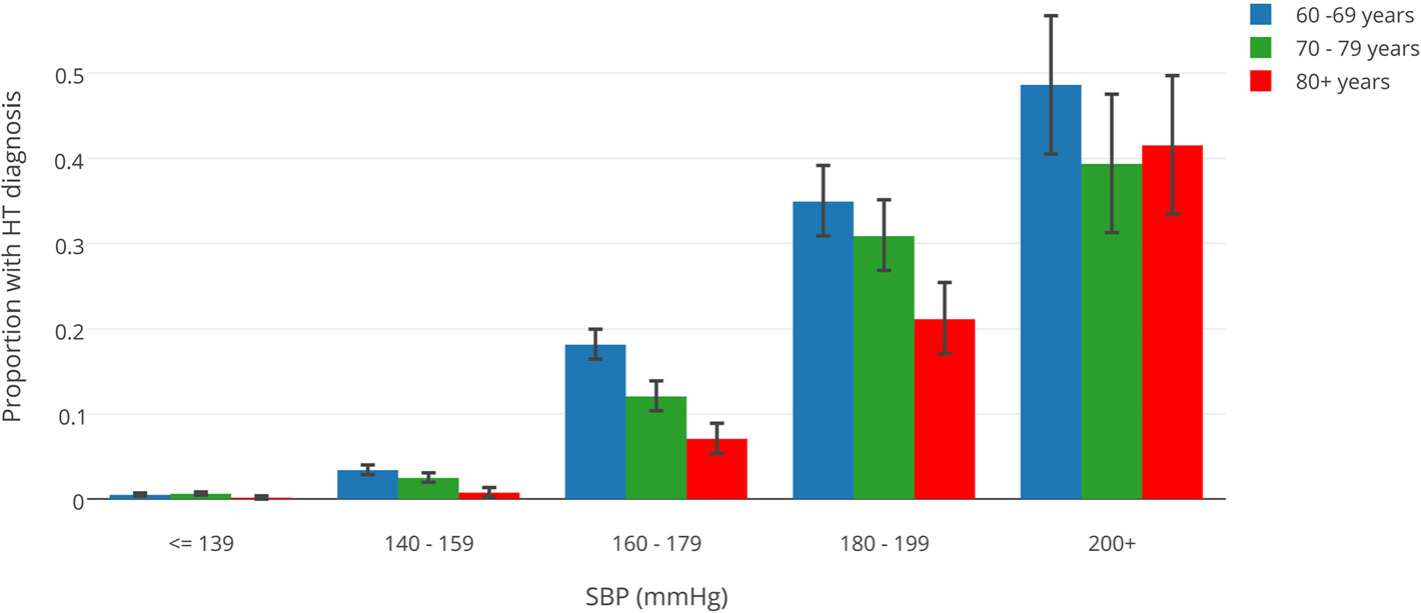
Observed proportions with hypertension diagnosis, by systolic blood pressure and age, with Wilson 95 % confidence intervals

Figure 1 shows the percentages of patients with a new diagnosis of HT for each age- and SBP-group with 95 % confidence intervals. The corresponding numbers are reported in Table 2.

**Table 2:** New diagnosis of hypertension within one month after inclusion

SBP (mmHg)	Age (years)	n	New Diagnosis (%)	p for trend
<= 130	60–69	5385	0.5 (0.3–0.7)	0.61
	70–79	1965	0.6 (0.3–1.1)	
	80+	661	0.2 (0.0–0.9)	
140–159	60–69	4029	3.4 (2.9–4.0)	<0.001
	70–79	2019	2.5 (1.9–.3.2)	
	80+	807	0.7 (0.3–1.6)	
160–179	60–69	1829	18.2 (16.5–20.0)	<0.001
	70–79	1028	12.1 (10.2–14.2)	
	80+	424	7.1 (5.0–9.9)	
180–199	60–69	504	34.9 (30.9–39.2)	0.002
	70–79	392	30.9 (26.5–35.6)	
	80+	156	21.2 (15.5–28.2)	
200+	60–69	142	48.6 (40.5–56.7)	0.24
	70–79	94	39.4 (30.1–49.5)	
	80+	65	41.5 (30.4–53.7)	

Percentage with Wilson 95 % CI by age group and blood pressure group; p-values for linear by linear association between age groups

Of those patients with a BP between 160 and 179 mmHg and aged 60–69 years, 18.2 % (16.5–20.0) had a new diagnosis of HT within 1 month. For patients of 70–79 years and 80 years and older within the same BP group these percentages were 12.1 % (10.2–14.2) and 7.1 % (5.0–9.9) respectively.

The p-value for trends between age groups was significant in all three BP groups between 140 and 200 mmHg, indicating that, within these SBP groups, older patients are less likely to be diagnosed as hypertensive than younger patients.

### Effect of systolic blood pressure Z-scores, age and gender on new diagnosis of hypertension

The outcomes of the logistic regression analysis with newly diagnosed HT as outcome were as follows. The unadjusted odds ratio (OR) for the SBP Z-score was 4.30 (95 % CI 4.01 – 4.61; *p* < 0.001). Adjusted for age, the OR for the SBP Z-score was 4.35 (4.05–4.67; *p* < 0.001).

This adjusted OR of 4.35 for SBP Z-score means that the odds of new diagnosis of HT is multiplied by 4.35 when the SBP increases by one standard deviation. Adjusted for SBP Z-score, age had a significant effect on the probability of diagnosis of HT (OR 0.98 per year age increase, 95 % CI 0.97–0.99, *p* > 0.001).

We found no significant interaction between age and SBP Z-score in our model. Gender had no significant contribution to the model.

This model permits calculating the probability of a new diagnosis of HT on basis of a given SBP Z value and age. For example: a 60-year-old male patient with a SBP Z-score +1 (165 mmHg) has a predicted probability of a new diagnosis of 15.9 % (95 % CI: 14.5–17.3 %). The probability for an 80-year-old with the same SBP Z-score (170 mmHg), is only 11.2 % (95 % CI: 10.1–12.5 %).

**Fig. 2:**
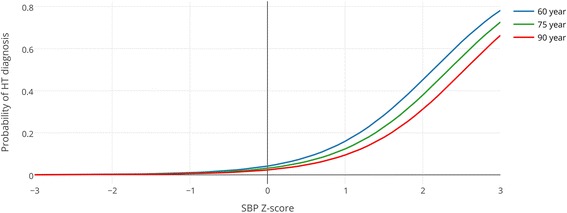
Predicted probability of hypertension diagnosis, by systolic blood pressure Z-score and age; based on logistic regression analysis

These findings are illustrated in Fig. 2 which shows the predicted probabilities of a new HT diagnosis, in relation to SBP and age.

### Percentages of patients with treatment of hypertension, by age and systolic blood pressure

We calculated the percentages of patients that received antihypertensive treatment within 4 months after being diagnosed as having HT. In the group with an SBP of 140–159 mmHg for instance, 45.3 % (37.2–53.6) of the 60–69 years old patients and 50.0 % (18.8–81.8) of the 80 years and older patients were treated; and in the group with an SBP of 160–179 mmHg, 69.9 % (64.7–74.6) of the 60–69 years old patients and 50.0 % (33.2–66.8) of the 80 years and older patients were treated. For details on all age groups please see Table 3. None of the p-values for age-related trends were significant.

**Table 3:** Treatment within four months after inclusion in diagnosed hypertension patients

SBP (mmHg)	Age (years)	N	Antihypertensive treatment (%)	p for trend
<=139	60–69	26	30.8 (16.5–50.0)	0.70
	70–79	12	33.3 (13.8–60.9)	
	80+	1	0.0 (0.0–79.3)	
140–159	60–69	137	45.3 (37.2–53.6)	0.70
	70–79	50	48.0 (44.8–62.5)	
	80+	6	50.0 (18.8–81.8)	
160–179	60–69	332	69.9 (64.7–74.6)	0.15
	70–79	124	71.0 (62.4–78.2)	
	80+	30	50.0 (33.2–66.8)	
180–199	60–69	176	86.9 (81.2–91.1)	0.25
	70–79	121	81.8 (74.0–87.7)	
	80+	33	81.8 (65.6–91.4)	
200+	60–69	69	95.7 (88.0–98.5)	0.28
	70–79	37	97.3 (86.2–99.5)	
	80+	27	88.9 (71.9–96.1)	

Percentage with Wilson 95 % CI by age group and blood pressure group; p-values for linear by linear association between age groups

### Effect of systolic blood pressure Z-scores, age and gender on treatment after diagnosis of hypertension

For the outcome treatment, the OR for the SBP Z-score was 2.96 (95 % CI: 2.45–3.57).

Age and gender had no significant contribution to the model. (OR for age 1.00, 95 % CI: 0.98–1.02, *p* = 0.82).

## Discussion

### Strengths and limitations

The study included a large and, because we excluded patients with diabetes, existing HT and other cardiovascular conditions, homogenous cohort. Moreover, the data were collected during the normal practice of GPs, and reflect how they manage HT in daily care. We have no reason to assume that GPs participating in IPCI differ in their HT management from their non-participating colleagues; therefore, the conclusions may be generalized to the Dutch population.

In the study we only studied recorded BP measurements and diagnoses. Especially diagnoses by medical specialists may not all be recorded by the GP. Therefore, registration bias is possible. All patients in our study have an immortal time of two years, which may introduce age related bias, as older patients have a shorter life expectancy and hence are less likely to be included in the cohort. Moreover, in general, diagnosis of HT is based on more than one measurement, which is not accounted for in our study. This may further explain why the percentages with a diagnosis of HT are rather low. A further limitation may be that this study does not permit any insight into the reason why older patients are less likely to be diagnosed as having HT.

For our study we excluded patients with co-morbidities such as DM and pre-existing cardiovascular disease. On the one hand this reduces the generalizability of the results. On the other hand, excluding patients with these co-morbidities eliminates these co-morbidities as potential confounders because, according to international guidelines, these patients should have strict BP control. By excluding these patients, it was easier to study the impact of age and BP on the outcomes. A strength of this study is that, by using reference SBP values we could control for the natural increase of BP by age. As reference BP data for the older Dutch population were are not readily available, we used data from the North-western Europe population, which may be a limitation. In our cohort, mean BPs in all age strata were slightly lower than the used reference values [16]. This may be explained by the fact that we excluded patients already diagnosed with HT.

### Comparison with existing literature

Although previous studies compared HT management in older to younger age groups [11, 12], to our knowledge, this is the first study to take age-specific SBP levels into account.

The proportions of patients with a new diagnosis of HT were rather low and this seems in accordance with existing literature about guideline adherence [10]. However, our follow up period of one month does not allow conclusions about guideline adherence as some patients may receive the diagnosis of HT later on.

Although we demonstrate that older patients are less likely to receive a diagnosis of HT, our data do not provide any insight why these patients are less likely to be diagnosed. A possible explanation may be the clinical condition, especially frailty in older patients as an important reason to refrain from treatment of HT [3, 9]. Other barriers to guideline compliance in older people are outside the scope of this study.

## Conclusions

This study showed that GPs are less inclined to diagnose HT with increasing age, but do not withhold treatment when they diagnose HT in older patients.

Increasing life expectancy will lead to larger numbers of older patients, yet the optimal management of HT in this age group has not been elucidated. In this respect there are indications that BP target values will depend on features, such as frailty, in older patients.

More research, especially in the form of trials, will be needed to determine whether the Dutch GP’s reservations to diagnose HT in older patients are correct.

## Appendix 1

Inclusion criteria based on the presence of ICPC codes. Diagnosis of HT was based on the presence of the ICPC code: K86 ‘hypertension without organic damage’. Diagnosis of atherosclerotic cardiovascular disease was based on the presence of the following ICPC codes:K87 'hypertension with organic damage', K74 'angina pectoris', K75 'acute myocardial infarction', K76 'other chronic cardiovascular disease', K77 'cardiac failure', K78 'atrial fibrillation', K89 'transient ischemic attack', K90 cerebrovascular disease, K9l 'atherosclerosis', K92.01 'intermittent claudication', and K99.01 'aortic aneurysm'.

## Appendix 2

Classification criteria for antihypertensive medication, based on ATC codes. The Anatomical Therapeutic Chemical (ATC) Classification System divides medication into groups, according to organ system and mode of action. Prescriptions of antihypertensive medication within the follow up period was based on the presence of one or more of the following ATC codes:

1. low ceiling diuretics (ATC C03A, C03B, C03E, C07B, C07D, C09BA and C09DA)
2. beta blockers (C07)
3. RAAS inhibitors (C09)
4. calcium channel blockers with mainly vascular effects (C08C, C09BB, C09BB)

## Abbreviations

ATC, anatomical therapeutic chemical; BP, blood pressure; DM, diabetes mellitus; ESC, European Society of Cardiology; ESH, European Society of Hypertension; GP, general practitioner; HT, hypertension; HYVET, hypertension in the very elderly trial; ICPC, International Classification of Primary Care; IPCI, Integrated Primary Care Information; NICE, British National Institute for Health and Care Excellence; OR, odds ratio; RAAS, Renin-Angiotensin-Aldosterone System; SBP, systolic blood pressure; WHO, World Health Organization
